# Formulation and Evaluation of Transdermal Patch of Repaglinide

**DOI:** 10.5402/2011/651909

**Published:** 2011-07-20

**Authors:** Shailesh T. Prajapati, Charmi G. Patel, Chhagan N. Patel

**Affiliations:** Department of Pharmaceutics, Shri Sarvajanik Pharmacy College, Gujarat, Mehsana 384001, India

## Abstract

Repaglinide has the half life of 1 hour, and bioavailability in the body is 56% due to first-pass metabolism. The total daily dose of Repaglinide is 16 mg (e.g., 4 mg four times daily depending on meal patterns); hence, it required frequent dosing. Transdermal patch of Repaglinide was prepared to sustain the release and improve bioavailability of drug and patient compliance. Different formulations were prepared by varying the grades of HPMC and concentration of PVP K30 by solvent casting method. The prepared formulations were evaluated for various parameters like thickness, tensile strength, folding endurance, % elongation, % moisture content, % moisture uptake, % drug content, in vitro drug release, in vitro permeation, and drug excipient compatibility. A 3^2^ full factorial design was applied to check the effect of varying the grades of HPMC (*X*_1_) and PVP concentration (*X*_2_) on the responses, that is, tensile strength, percentage drug released in 1 hr (*Q*_1_), 9 hr (*Q*_9_), and diffusion coefficient as a dependent variables. *In vitro* release data were fitted to various models to ascertain kinetic of drug release. Regression analysis and analysis of variance were performed for dependent variables. The results of the F2 statistics between factorial design batches and theoretical profile were used to select optimized batch. Batch F6 was considered optimum batch which contained HPMC K100 and PVP (1.5%), showed release 92.343% up to 12 hr, and was more similar to the theoretical predicted dissolution profile (*f*_2_ = 69.187).

## 1. Introduction

Transdermal drug delivery system (TDDS) has been an increased interest in the drug administration via the skin for both local therapeutic effects on diseased skin (topical delivery) as well as for systemic delivery of drugs. The skin as a site of drug delivery has a number of significant advantages over many other routes of drug administration, including the ability to avoid problems of gastric irritation, pH and emptying rate effects, avoid hepatic first-pass metabolism thereby increasing the bioavailability of drug, reduce the risk of systemic side effects by minimizing plasma concentrations compared to oral therapy, provide a sustained release of drug at the site of application; rapid termination of therapy by removal of the device or formulation, the reduction of fluctuations in plasma levels of drugs, and avoid pain associated with injections. The transdermal delivery can also eliminate pulsed entry into the systemic circulation, which might often cause undesirable side effects [1]. 

Diabetes mellitus is a major and growing health problem worldwide and an important cause of prolonged ill health and early death. It is a chronic metabolic disorder characterized by a high blood glucose concentration (hyperglycemia) caused by insulin deficiency, and it is often combined with insulin resistance [2]. Repaglinide is an oral blood-glucose-lowering drug of the meglitinide class use to treat NIDDM (noninsulin-dependent diabetes mellitus). It lowers blood glucose by stimulating the release of insulin from the pancreas. It has an extremely short half life of 1 h. In addition, the oral bioavailability of Repaglinide is low (56%) due to extensive hepatic first-pass effect. Dosage frequency of Repaglinide is 0.5 to 4 mg in 3 to 4 times in a day. It has melting point of 130-131°C and mol. wt. 452.58 [3–6]. It belongs to class 2 drug. Repaglinide topical preparation may be beneficial to the patient since it reduce adverse effects and avoid the hepatic first-pass metabolism. The need for transdermal delivery of Repaglinide is further justified due to the requirement of maintaining unfluctuating plasma concentrations for effective management of blood sugar for long period in diabetic patients.

The purpose of the present work was to develop transdermal formulation of Repaglinide which increases the patient compliance and also sustain the release of drug to increase the bioavailability by using different grades of HPMC and PVP K30 as polymers.

## 2. Materials and Methods

### 2.1. Materials

Repaglinide was received as a gift sample from Torrent Pharmaceutical Ltd., Gujarat, India. HPMC K4M, HPMC K100, and HPMC E15 LV were purchased from Yarrow Chem, Mumbai, India. PVP K30 was obtained from SD fine—Chem. Ltd, Mumbai. Polyethylene glycol 400 and propylene glycol were obtained from Merck Specialities Private Ltd. (Mumbai) and Chemdyes Corporation (Ahmedabad, Gujarat) respectively. Cellulose acetate membrane was obtained from Sartorius Biotech GmbH (Germany). All other materials and chemicals used were of either pharmaceutical or analytical grade.

### 2.2. Methods

#### 2.2.1. Preparation of Transdermal Patch

Drug-loaded matrix-type transdermal patches of Repaglinide were prepared by using solvent casting method. A petri dish with a total area of 44.15 cm^2^ was used. Polymers were accurately weighed and dissolved in 10 mL of water, methanol (1 : 1) solution and kept aside to form clear solution. Drug was dissolved in the above solution and mixed until clear solution was obtained. Polyethylene glycol 400 (30% w/w of total polymer) was used as plasticizer and propylene glycol (15% w/w of total polymer) was used as permeation enhancer. The resulted uniform solution was cast on the petri dish, which was lubricated with glycerin and dried at room temperature for 24 h. An inverted funnel was placed over the petri dish to prevent fast evaporation of the solvent. After 24 h, the dried patches were taken out and stored in a desiccator for further studies [7].

#### 2.2.2. Preliminary Screening

Preliminary study was carried out to check effect of various polymer combinations on transdermal patch formulation. Composition of preliminary trial batches P1 to P5 is shown in Table 1.

#### 2.2.3. Optimization of Variables Using Full Factorial Design

A 3^2^-randomized full factorial design was used in the present study. In this design, 2 independent factors were evaluated, each at 3 levels, and experimental trials were performed for all 9 possible combinations. The different grades of HPMC (*X*_1_) and concentration of PVP K30% (*X*_2_) were chosen as independent variables in 3^2^ full factorial designs. Tensile strength, cumulative % drug release at 1 h (*Q*_1_), cumulative % drug release at 9 h (*Q*_9_), and diffusion coefficient (*n*) were taken as dependent variables. The formulation layout for the factorial design batches (F1 to F9) are shown in Table 2.

#### 2.2.4. Evaluation Parameters of Transdermal Patch

**Folding Endurance [8]**

A strip of specific area (2 cm*2 cm) was cut evenly and repeatedly folded at the same place till it broke. The number of times the film was folded at the same place without breaking gave the value of the folding endurance.

**Tensile Strength [7]**

The tensile strength of the patch was evaluated by using the tensiometer (Erection and instrumentation, Ahmedabad). It consists of two load cell grips. The lower one was fixed and upper one was movable. Film strips with dimensions of 2*2 cm were fixed between these cell grips, and force was gradually applied till the film broke. The tensile strength was taken directly from the dial reading in kg.

**Percentage Elongation Break Test [9]**

The percentage elongation break was determined by noting the length just before the break point, the percentage elongation was determined from the below mentioned formula.

(1) $$\text{Elongation percentage}\;=\;\left[\frac{\left(L_{1}-L_{2}\right)}{L_{2}}\right]\;\;\times 100,$$

where *L*_1_ is the final length of each strip, and *L*_2_ is the initial length of each strip.

**Thickness [8]**

Patch thickness was measured using digital micrometer screw gauge at three different places, and the mean value was calculated.

**Drug Content [1]**

A specified area of patch (2 cm*2 cm) was dissolved in 100 mL methanol and shaken continuously for 24 h. Then the whole solution was ultrasonicated for 15 min. After filtration, the drug was estimated spectrophotometrically at wavelength of 281 nm and determined the drug content.

**Percentage Moisture Content [8]**

The prepared films were weighed individually and kept in a desiccator containing fused calcium chloride at room temperature for 24 h. After 24 h, the films were reweighed and determined the percentage moisture content from the below mentioned formula:

(2) $$\begin{array}{cc} & \text{Percentage moisture content}\\ & \;\;=\left[\frac{\left(\text{Initial weight}-\text{Final weight}\right)}{\text{Final weight}}\right]\times 100. \end{array}$$

**Percentage Moisture Uptake [8]**

The weighed films were kept in a desiccators at room temperature for 24 h containing saturated solution of potassium chloride in order to maintain 84% RH. After 24 h, the films were reweighed and determine the percentage moisture uptake from the below mentioned formula:

(3) $$\begin{array}{cc} & \text{Percentage moisture uptake}\\ & \;\;=\left[\frac{\left(\text{Final weight}-\text{Initial weight}\right)}{\text{Initial weight}}\right]\times 100. \end{array}$$

***In Vitro* Drug Release Studies [1]**

*In Vitro *drug release studies were performed by using a Franz diffusion cell with a receptor compartment capacity of 60 mL. The cellulose acetate membrane was used for the determination of drug from the prepared transdermal matrix-type patches. The cellulose acetate membrane having a pore size 0.45 *μ* was mounted between the donor and receptor compartment of the diffusion cell. The prepared transdermal film was placed on the cellulose acetate membrane and covered with aluminum foil. The receptor compartment of the diffusion cell was filled with phosphate buffer pH 7.4. The whole assembly was fixed on a hot plate magnetic stirrer, and the solution in the receptor compartment was constantly and continuously stirred using magnetic beads, and the temperature was maintained at 32 ± 0.5°C, because the normal skin temperature of human is 32°C. The samples were withdrawn at different time intervals and analyzed for drug content spectrophotometrically. The receptor phase was replenished with an equal volume of phosphate buffer at each sample withdrawal.

***In Vitro *Permeation Study [10]**

An in vitro permeation study was carried out by using Franz diffusion cell. Full thickness abdominal skin of male Wistar rat weighing 200 to 250 g was used. Hair from the abdominal region was removed carefully by using an electric clipper; the dermal side of the skin was thoroughly cleaned with distilled water to remove any adhering tissues or blood vessels, equilibrate for an hour in phosphate buffer pH 7.4 before starting the experiment, and was placed on a magnetic stirrer with a small magnetic needle for uniform distribution of the diffusant. The temperature of the cell was maintained at 32 ± 0.5°C using a thermostatically controlled heater. The isolated rat skin piece was mounted between the compartments of the diffusion cell, with the epidermis facing upward into the donor compartment. Sample volume of 5 mL was removed from the receptor compartment at regular intervals, and an equal volume of fresh medium was replaced. Samples were filtered through watman filter and were analyzed using Shimadzu UV 1800 double-beam spectrophotometer (Shimadzu, Kyoto, Japan). Flux was determined directly as the slope of the curve between the steady-state values of the amount of drug permeated (mg*cm^2^) versus time in hours and permeability coefficient was deduced by dividing the flux by the initial drug load (mg*cm^2^).

#### 2.2.5. Kinetic Modeling of Dissolution Data

The release profile of all batches were fitted to various mathematical models such as Zero order, First order, Higuchi [11], Hixon and Crowell [12], and Korsmeyer et al. [13], to ascertain the kinetic of drug release.

#### 2.2.6. Comparison of Dissolution Profiles for Selection of Optimum Batch

The similarity factor (*f*_2_) given by SUPAC guidelines for a modified release dosage form was used as a basis to compare release profiles. The release profiles are considered to be similar when *f*_2_ is between 50 and 100. The release profile of products were compared using an *f*_2_ which is calculated from following formula:

(4) $$f_{2}=50\times \log\left\{{\left[1+\left(\frac{1}{n}\right)\overset{n}{\underset{t=1}{\sum }}w_{t}{\left(R_{t}-T_{t}\right)}^{2}\right]\;}^{-0.5}\times 100\right\},$$

where *n* is the release time and *R*_*t*_ and *T*_*t*_ are the reference (here this is the theoretical profile of Repaglinide and test value at time *t* [14].

#### 2.2.7. Drug Excipients Compatibility Study

Fourier transform infrared (FTIR) technique was used to study the physical and chemical interaction between drug and excipients. FTIR spectrum of Repaglinide, HPMC K4M, HPMC K100, HPMC E15 LV, PVP K30, and a physical mixture of Repaglinide: HPMC (K100/K4M/E15 LV): PVP K30 was recorded using KBr mixing method on FTIR (FTIR-1700, Shimadzu, Kyoto, Japan). IR spectra are shown in Figures 7 and 8

## 3. Result and Discussion

### 3.1. Preliminary Study

All the batches of transdermal patch showed thickness variation range from 0.12 to 0.20 mm as shown in Table 3. High thickness of batch P4 and P5 was found, it may be due to low solubility of ethyl cellulose in solvent render uneven distribution of polymer layer. All the batches of transdermal patch showed tensile strength and % elongation in uniform range from 16 to 22 and 17.5 to 22.5, respectively, except batches P4 and P5 may be due to poor solubility of ethyl cellulose and weak bond formation (Table 3). Hence batches P4 and P5 were eliminated for further study. Batch P1 containing PVA : PVP shows fast release of drug (101.26% at 8 h) from patch due to burst effect of PVP and also more solubility in water. So batch P1 was also eliminated.

### 3.2. Folding Endurance, Tensile Strength, % Elongation and Thickness

The results of folding endurance, tensile strength, % elongation and thickness of factorial design batches are shown in Table 4. The folding endurance values of all the factorial design patches were found satisfactory which indicates that the patches prepared using PEG 400 in a concentration of 30% w/w of polymer were having optimum flexibility and were not brittle. The tensile strength of the patches prepared with HPMC E15 and PVP were found in between 0.38 ± 0.015 kg/cm^2^ to 0.63 ± 0.015 kg/cm^2^, which were 0.45 ± 0.014 kg/cm^2^ to 0.92 ± 0.017 kg/cm^2^ for the patches composed of HPMC K100 and were 0.53 ± 0.011 kg/cm^2^ to 0.95 ± 0.015 kg/cm^2^ for the patches composed of HPMC K4M. It was observed that with the increase of PVP concentrations and HPMC grade, the tensile strength of the patches gradually increased. The % elongation was found to be in the range of 28.95 ± 0.015% to 41.2 ± 0.015%. The formulation F8 showed minimum % elongation among the other entire factorial design batches 28.95 ± 0.015%. It indicates inverse relation between tensile strength and % elongation. The thickness ranges were 0.12 ± 0.025 to 0.25 ± 0.022 mm. The results showed that the patches were uniform, as it was evidenced by SD value, which were less than 0.01 for all the factorial design batches.

### 3.3. Moisture Content, Moisture Uptake, and Drug Content Studies

The moisture content in the patches ranged from 3.24 ± 0.017 to 4.12 ± 0.015%. The moisture content in the formulations was found to be increased by increase in the concentration of PVP K30 and also with increasing the grade of HPMC. The moisture uptake in the patches ranged from 5.27 ± 0.012 to 7.89 ± 0.019%. The moisture uptake was found to be higher in batches F7, F8, and F9, which might be due to HPMC K4M. The lower moisture content in the formulations helps them to remain stable and become a completely dried and brittle film. Again, low moisture uptake protects the material from microbial contamination and bulkiness. The drug content ranged from 74.282 to 78.98%. All formulations were acceptable with regard to Repaglinide content (Table 4).

### 3.4. In Vitro Drug Release Study

**Figure 1:**
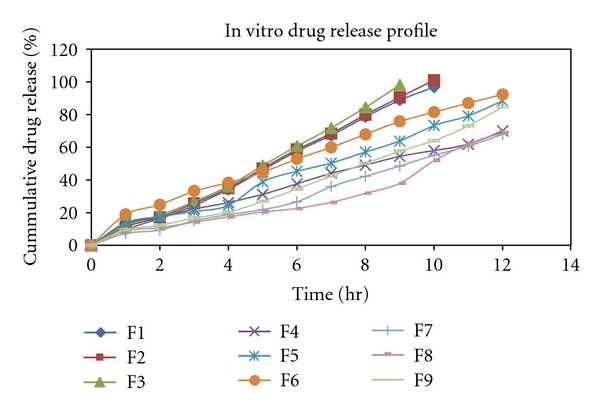
In vitro drug release profile for batch F1 to F9.

The drug release characteristics of the formulation were studied in *in vitro *conditions by using artificial semipermeable membrane. The formulation F1–F3 has shown release of about 96.83%, 101.057% at 10 h and 98.26% at 9 h, respectively. This is may be due to low viscosity of HPMC E15 LV polymer which is rapidly soluble than HPMC K4M and HPMC K100. The formulation F4–F9 has shown release of about 70.02%, 88.49%, 92.343%, 68.01%, 69.014%, and 84.804% at 12th hour, respectively (Figure 1). HPMC K4M shows slow release of drug from patch due to matrix formation and also its high viscosity which affect the release while HPMC K100 shows predicted release. The order of drug release was found to be F2>F3>F1>F6>F5>F9>F4>F8>F7. The in vitro release data of F1 to F7 formulations fitted well into the Zero order equation, correlation coefficient values were between 0.9869 and 0.9986 while F8 and F9 follows first-order release. Hixon crowell law and Highuchi model was applied to test the release mechanism. *R* ^2^ values are higher for Highuchi model than Hixon crowell for all formulations, hence, drug release from all batches follow diffusion rate-controlled mechanism. According to Korsmeyer-Peppas model, a value of slope for F1, F2, F3, F7, and F9 was >0.85, so it indicates that the release mechanism follows zero order while for F4, F5, F6, and F8 was between 0.5 to 0.85 which indicates the release mechanism was non-Fickian diffusion (Table 2) [15].

### 3.5. In Vitro Permeation Study

**Figure 2:**
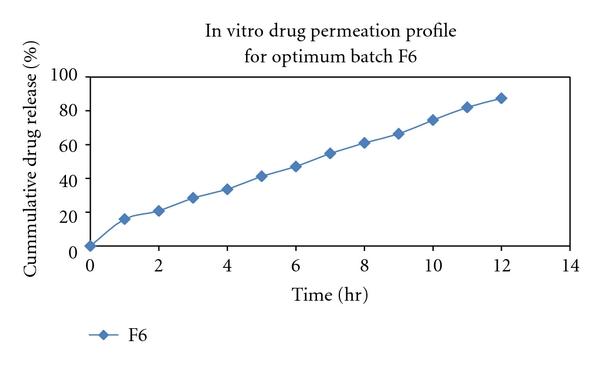
*In vitro* drug permeation profile for batch F6.

**Figure 3:**
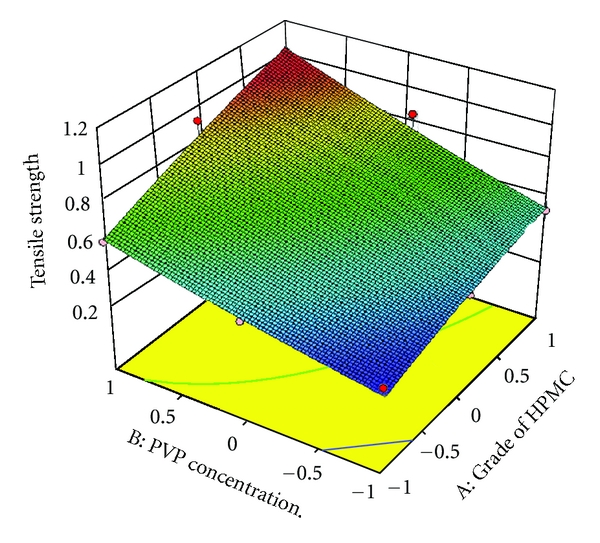
Response surface plot for tensile strength.

The formulation F6 exhibited 87.4% of drug permeated in 12 h with a flux of 8.65 *μ*g/cm^2^/h (with a permeation coefficient of 3.967 cm/h) (Figure 2). Plotting the cumulative amounts of drug permeated per square centimeter of the patches through the rat abdominal skin against time showed that the permeation profiles of drug might follow zero-order kinetics as it was evident by correlation coefficients 0.992, better fit than first order (*R* ^2^ = 0.982) and Higuchi model (*R* ^2^ = 0.987) (Figure 3). According to korsmeyer-Peppas model, a value of slope for F6 was between 0.5 and 0.85 (0.678) which indicates that the release mechanism was non-Fickian diffusion. The results of drug permeation from transdermal patches of Repaglinide through the rat abdominal skin confirmed that Repaglinide was released from the formulation and permeated through the rat skin and, hence, could possibly permeate through the human skin.

### 3.6. Full Factorial Design

A statistical model incorporating interactive and poly nominal terms was used to evaluate the responses.

(5) $$Y_{i}=b_{0}+b_{1}X_{1}+b_{2}X_{2}+b_{12}X_{1}X_{2}+b_{11}X_{1}^{2}+b_{22}X_{2}^{2},$$

where *Y* is the dependent variable, *b*_0_ is the arithmetic mean response of the 9 runs, and *b*_*i*_ is the estimated coefficient for the factor *Xi*. The main effects (*X*_1_ and *X*_2_) represent the average result of changing 1 factor at a time from its low to high values. The two way interaction terms (*X*_12_) show how the response changes when two factors are simultaneously changed. Polynomial terms (*X*_11_ and *X*_22_) are included to investigate nonlinearity. The in vitro release profile for 9 batches showed a variation (i.e., tensile strength, % cumulative drug release at 1 h (*Q*_1_), % cumulative drug release at 9 h (*Q*_9_), and diffusion coefficient). The data indicate that the release profile of the drug is strongly dependent on the selected independent variables. The fitted equations (full and reduced) relating the responses, tensile strength, *Q*_1_, *Q*_9_, and diffusion coefficient to the transformed factor are shown in Table 5. The polynomial equations can be used to draw conclusions after considering the magnitude of coefficient and the mathematical sign it carries (i.e., negative or positive).  Table 6 shows the results of analysis of variance (ANOVA), which was performed to identify insignificant factors. Data were analyzed using Microsoft Excel. 

*R* ^2^ value for tensile strength, *Q*_1_, *Q*_9_, and diffusion coefficient are 0.9431, 0.9318, 0.9648, and 0.8030, respectively, indicating good correlation between dependent and independent variables. The reduced models were developed for response variables by omitting the insignificant terms with *P* > 0.05. The terms with *P* < 0.05 were considered statistically significance and retained in the reduced model. The coefficients for full and reduced models for response variables are shown in Table 5.

### 3.7. Full and Reduced Model for Tensile Strength

The significance levels of the coefficients *b*_12_, *b*_1_^2^ and *b*_2_^2^ were found to be *P* = 0.3833, 0.7763, and 0.7959, respectively; hence, they were omitted from the full model to generate a reduced model. The results of statistical analysis are shown in Table 5. The coefficients *b*_1_ and *b*_2_ were found to be significant at *P* < 0.05; hence, they were retained in the reduced model. The reduced model was tested in proportion to determine whether the coefficients *b*_12_,  *b*_1_^2^, and *b*_2_^2^ contribute significant information to the prediction of tensile strength. The results of model testing are shown in Table 6. The critical value of *F* for *α* = 0.05 is equal to 9.27 (df = 3,3). Since the calculated value (*F* = 0.404) is less than critical value (*F* = 9.27), it may be concluded that the terms *b*_12_, *b*_1_^2^, and *b*_2_^2^ do not contribute significantly to the prediction of tensile strength and can be omitted from the full model to generate the reduced model.

### 3.8. Full and Reduced Model for *Q*_1_

The significance levels of the coefficients *b*_1_, *b*_2_, *b*_12_ and *b*_2_^2^ were found to be *P* = 0.129, 0.064, 0.962, and 0.658, respectively, so they were omitted from the full model to generate a reduced model. The results of statistical analysis are shown in Table 5. The coefficient *b*_1_^1^ was found to be significant at *P* < 0.05; hence, it was retained in the reduced model. The reduced model was tested in proportion to determine whether the coefficient *b*_1_, *b*_2_, *b*_12_, and *b*_2_^2^ contribute significance information to the prediction of *Q*_1_. The results of model testing are shown in Table 6. The critical value of *F* for *α* = 0.05 is equal to 9.11 (df = 4,3). Since the calculated value (*F* = 3.209) is less than critical value (*F* = 9.11), it may be concluded that the term *b*_1_, *b*_2_, *b*_12_, and *b*_2_^ 2^ do not contribute significantly to the prediction of *Q*_1_ and can be omitted from the full model to generate the reduced model.

### 3.9. Full and Reduced Model for *Q*_9_

The significance levels of the coefficients *b*_2_, *b*_12_, *b*_1_^2^, and *b*_2_^2^ were found to be *P* = 0.0849, 0.9905, 0.3062, and 0.2466, respectively, so they were omitted from the full model to generate a reduced model. The results of statistical analysis are shown in Table 5. The coefficient *b*_1_ was found to be significant at *P* < 0.05; hence, it was retained in the reduced model. The reduced model was tested in proportion to determine whether the coefficient *b*_2_, *b*_12_, *b*_1_^2^, and *b*_2_^2^ contribute significance information to the prediction of *Q*_9_. The results of model testing are shown in Table 6. The critical value of F for *α* = 0.05 is equal to 9.11 (df = 4,3). Since the calculated value (*F* = 2.50) is less than critical value (*F* = 9.11), it may be concluded that the term *b*_2_, *b*_12_, *b*_1_^2^ and *b*_2_^2^ do not contribute significantly to the prediction of *Q*_9_ and can be omitted from the full model to generate the reduced model.

### 3.10. Full and Reduced Model for Diffusion Coefficient

The results of statistical analysis are shown in Table 5. None of the coefficients were found to be significant at *P* < 0.05; hence, reduced model was not obtained. So diffusion coefficient gives no significance effect. The results of model testing are shown in Table 6. The critical value of *F* for *α* = 0.05 is equal to 9.11 (df = 4,3). Since the calculated value (*F* = 3.09) is less than critical value (*F* = 9.11), it may be concluded that all the terms do not contribute significantly to the prediction of diffusion coefficient.

**Figure 4:**
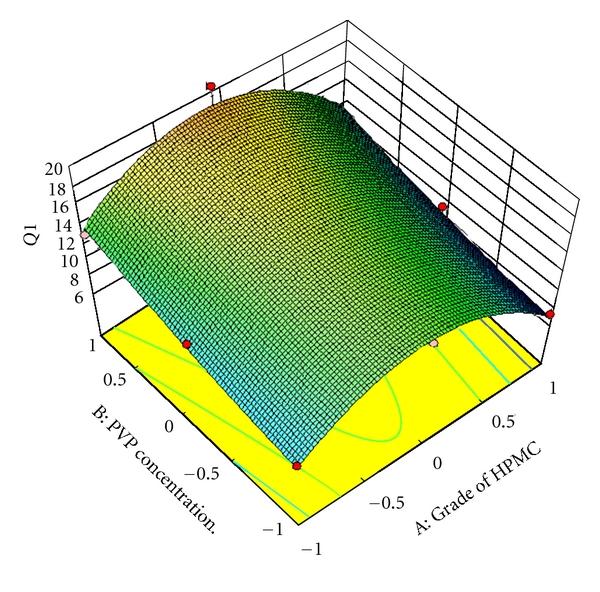
Response surface plot for *Q*_1_.

**Figure 5:**
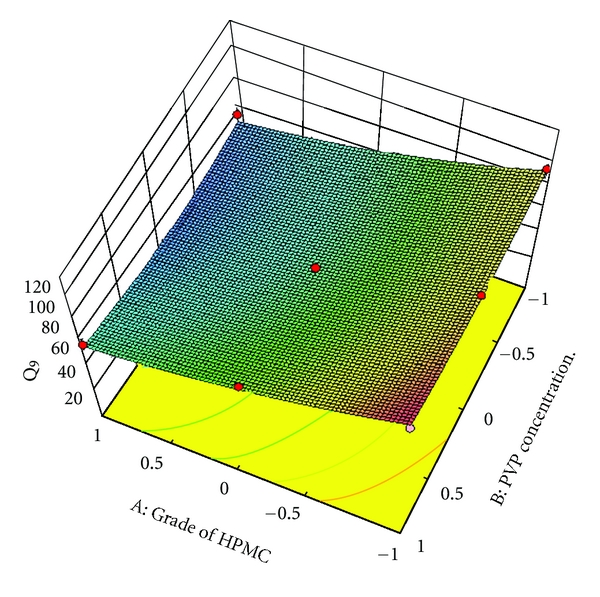
Response surface plot for *Q*_9_.

**Figure 6:**
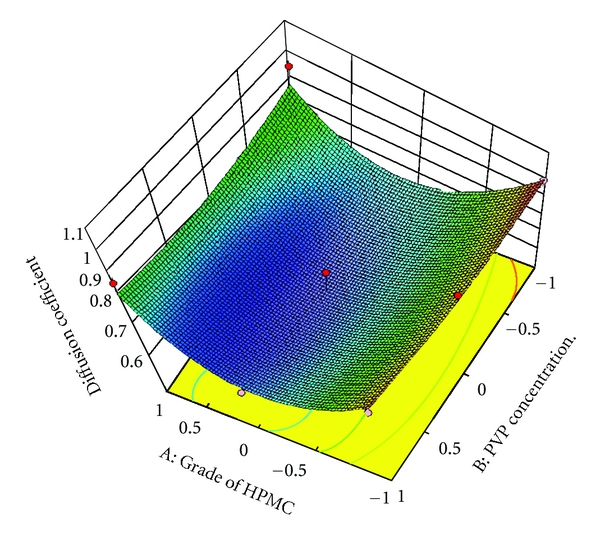
Response surface plot for diffusion coefficient.

To demonstrate graphically the effect of grade of HPMC and concentration of PVP K30, the response surface plots were generated by using Design expert 8.0.2 trial version software for the dependent variables tensile strength, *Q*_1_, *Q*_9_ (% drug release after 1, and 9 hours, resp.), and diffusion coefficient (*n*) shown in Figures 3–6, respectively.

### 3.11. Comparison of In Vitro Release Profile for Selection of Optimum Batch

Dissolution profiles of all batches of factorial design were compared with theoretical dissolution profile. The values of similarity factor (*f*_2_) for batches F1 to F9 are shown in Table 4. Batch F6 showed highest *f*_2_ value (69.187) among all the batches. Hence, batch F6 is more similar compared to other batches so it was selected as an optimum batch.

### 3.12. Drug Excipients Compatibility Study

**Figure 7:**
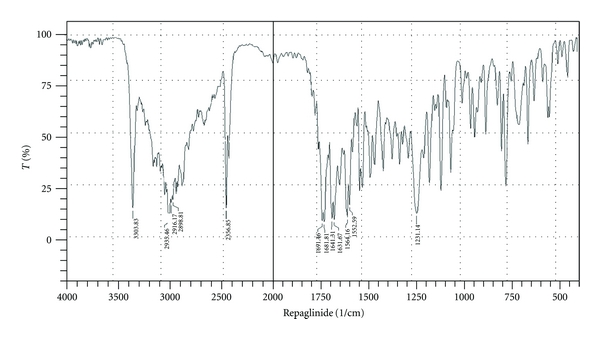
FTIR spectrum of Repaglinide.

**Figure 8:**
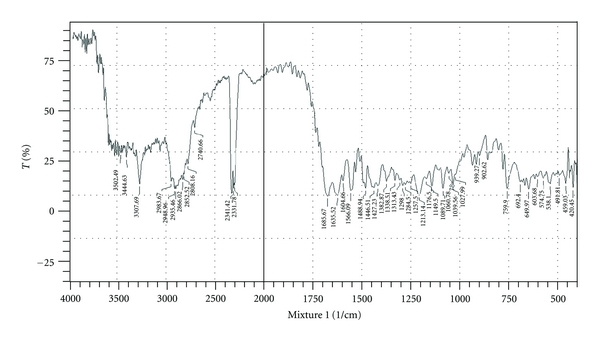
FTIR spectrum of optimized formulation composition.

Drug-excipients interactions play a vital role in the release of drug from formulation. The pure Repaglinide and its mixture with different grade of HPMC and PVP K30 were mixed separately with IR grade KBr and were scanned over a range of 400–4500 cm^−1^ using FTIR instrument (FTIR-1700, Shimadzu, Kyoto, Japan). The drug exhibits peaks due to ketonic group, alcohol group, secondary amine, terminal CH_3_ group, and C=O stretching in COOH and CONH. It was observed that main peaks of Repaglinide were present in mixture of drug and polymer, and no change in main peaks of the drug IR spectra in a mixture of drug and polymers was found. The FTIR study revealed no physical or chemical interactions of Repaglinide with each grade of HPMC and PVP K30 as evident from Figures 7 and 8.

## 4. Conclusion

The prepared transdermal drug delivery system of Repaglinide using different grades of HPMC and PVP K30 had shown good promising results for all the evaluated parameters. It was concluded that HPMC K100 and PVP K30 of moderate level useful for preparation of sustained release matrix transdermal patch formulation.

## Figures and Tables

**Table 1:** Preliminary trial batches.

Batch code	Polymer	Polymer proportion	Solvent	Plasticizer (30% w/w)*
P1	PVA : PVP	1 : 1	Water	PG
P2	HPMC K100M : PVP	1 : 1	Water	PEG
P3	HPMC K4M : PVP	1 : 1	Water	PEG
P4	EC : PVP	1 : 4	CHCl_3_	PG
P5	EC : HPMC K4M	3 : 7	Ethanol : DCM	PEG

Each batch contains 4 mg drug in 4 cm^2^ area. *30% w/w of total polymer weight.

**Table 2:** Formulation and evaluation of 3^2^ full factorial design batches.

Batch code	*X*1 (HPMC grade)	*X*2 (% PVP K30 concentration)	*Y*1 (tensile strength kg/cm^2^)	*Y*2 (*Q*_1_)(%)	*Y*3 (*Q*_9_)(%)	*Y*4 (diffusion coefficient)
F1	HPMC E15 LV	0.5	0.38	9.780612	88.91582	1.043
F2	HPMC E15 LV	1	0.46	11.31122	90.59949	1.001
F3	HPMC E15 LV	1.5	0.63	12.22959	98.26531	0.983
F4	HPMC K100	0.5	0.45	13.30102	54.54082	0.665
F5	HPMC K100	1	0.58	13.91327	63.78827	0.746
F6	HPMC K100	1.5	0.92	18.96429	75.90561	0.641
F7	HPMC K4M	0.5	0.53	7.331633	48.52041	0.959
F8	HPMC K4M	1	0.84	8.862245	37.98469	0.656
F9	HPMC K4M	1.5	0.95	9.627551	57.70408	0.853

**Table 3:** Results for preliminary trial batches.

Batch code	Thickness (mm)	Tensile strength (kg/cm^2^)	% elongation	Folding endurance	CPR (%)
P1	0.12	20	22.5	82	101.26
P2	0.13	22	22.5	79	90.03
P3	0.15	20	17.5	76	75.89
P4	0.15	9	5	22	65.27
P5	0.20	8	5	20	62.49

**Table 4:** Evaluation parameters of factorial batches F1 to F9.

Sr. no.	Batch code	Folding endurance	Tensile strength (kg/cm^2^) (mean ± S.D.)	% Elongation (Mean ± S.D.)	Thickness (mm) (mean ± S.D.)	% Drug content (mean ± S.D.)	*f*_2_ value
1	F1	<150	0.38 ± 0.015	41.2 ± 0.015	0.12 ± 0.025	78.98	52.58
2	F2	<150	0.46 ± 0.012	38.8 ± 0.014	0.15 ± 0.062	78.941	49.97
3	F3	>200	0.63 ± 0.015	37.1 ± 0.012	0.25 ± 0.022	78.63	45.44
4	F4	>200	0.45 ± 0.014	40.2 ± 0.013	0.22 ± 0.012	77.851	31.38
5	F5	>200	0.58 ± 0.015	39.6 ± 0.017	0.15 ± 0.015	78.327	44.75
6	F6	>200	0.92 ± 0.017	35.8 ± 0.012	0.17 ± 0.013	77.956	69.18
7	F7	>200	0.53 ± 0.011	39.2 ± 0.013	0.16 ± 0.021	75.829	26.13
8	F8	>200	0.84 ± 0.017	28.9 ± 0.015	0.13 ± 0.018	74.683	23.09
9	F9	>200	0.95 ± 0.015	30.1 ± 0.015	0.23 ± 0.015	74.282	34.03

Values expressed as mean ± S.D, *n* = 3.

**Table 5:** Summary of results of regression analysis.

Tensile strength						
Response	*b*_0_	*b*_1_	*b*_2_	*b*_12_	*b*_1_^2^	*b*_2_^2^
(tensile strength)						

FM	0.638889	0.141667	0.19	0.0425	−0.01833	0.016667
RM	0.637778	0.141667	0.19	–	–	–

*Q*1 hr						
Response (*Q*1)	*b*_0_	*b*_1_	*b*_2_	*b*_12_	*b*_1_^2^	*b*_2_^2^

FM	15.05272	−1.25	1.734694	−0.038	−5.53572	0.510204
RM	15.39286	–	–	–	−5.53572	–

*Q*9 hr						
Response (*Q*9)	*b*_0_	*b*_1_	*b*_2_	*b*_12_	*b*_1_^2^	*b*_2_^2^

FM	60.39966	−22.2619	6.649658	−0.041	5.586733	6.517858
RM	68.46939	−22.2619	–	–	–	–

Diffusion coefficient						
Response	*b*_0_	*b*_1_	*b*_2_	*b*_12_	*b*_1_^2^	*b*_2_^2^
(diffusion coefficient)						

FM	0.64641	−0.09322	−0.03172	−0.011	0.231885	0.056385
RM	–	–	–	–	–	–

FM: full model, RM: reduce model.

**Table 6:** Calculation for testing the model in portions.

Tensile strength						
	DF	SS	MS	*F*	*R* ^2^	
Regression						

FM	5	0.345469	0.069094	9.924378	0.94299	*F*_cal_ = 0.4047080856747338
RM	2	0.337017	0.168508	34.46109	0.91992	*F*_tab_ = 9.27663
Error						
FM	3	0.020886	0.006962	–	–	DF (3,3)
RM	6	0.029339	0.00489	–	–	

For *Q*1						
	DF	SS	MS	*F*	*R* ^2^	
Regression						

FM	5	89.24479	17.84896	8.196259	0.93179	*F*_cal_ = 3.209464
RM	1	61.28835	61.28835	12.43909	0.6399	*F*_tab_ = 9.11718
Error						
FM	3	6.533088	2.177696	–	–	DF (4,3)
RM	7	34.48953	4.927076	–	–	

For *Q*9						
	DF	SS	MS	*F*	*R* ^2^	
Regression						

FM	5	3386.258	677.2515	16.42181	0.964751	*F*_cal_ = 2.50177555838715
RM	1	2973.555	2973.555	38.80292	0.847171	*F*_tab_ = 9.11718
Error						
FM	3	123.723	41.24098	–	–	DF (4,3)
RM	7	536.4257	76.63224	–	–	

For Diffusion coefficient						
	DF	SS	MS	*F*	*R* ^2^	
Regression						

FM	5	0.172597	0.034519	2.445924	0.80302	*F*_cal_ = 3
RM	–	–	–	–	–	*F*_tab_ = 9.013455168
Error						
FM	3	0.042339	0.014113	–	–	DF (5,3)
RM	–	–	–	–	–	

*DF: degree of freedom; SS: sum of squares; MS: mean of squares; *R* ^2^: regression coefficient; FM: full model; RM: reduced model.
